# Anterior cruciate ligament microfatigue damage detected by collagen autofluorescence in situ

**DOI:** 10.1186/s40634-022-00507-6

**Published:** 2022-07-30

**Authors:** Jinhee Kim, So Young Baek, Stephen H. Schlecht, Mélanie L. Beaulieu, Lindsay Bussau, Junjie Chen, James A. Ashton-Miller, Edward M. Wojtys, Mark M. Banaszak Holl

**Affiliations:** 1 Department of Chemical & Biological Engineering, Monash University, Melbourne, Australia; 2 Department of Chemistry, University of Michigan, Ann Arbor, MI USA; 3 Department of Mechanical Engineering, University of Michigan, Ann Arbor, MI USA; 4 Department of Orthopaedic Surgery, Indiana University School of Medicine, Indianapolis, IN USA; 5 Department of Orthopaedic Surgery, University of Michigan, Ann Arbor, MI USA; 6 Optiscan, Mulgrave, VIC Australia

**Keywords:** Collagen autofluorescence, Confocal laser endomicroscopy, Anterior cruciate ligament, Microfatigue damage, Non-contact ACL injuries

## Abstract

**Purpose:**

Certain types of repetitive sub-maximal knee loading cause microfatigue damage in the human anterior cruciate ligament (ACL) that can accumulate to produce macroscopic tissue failure. However, monitoring the progression of that ACL microfatigue damage as a function of loading cycles has not been reported. To explore the fatigue process, a confocal laser endomicroscope (CLEM) was employed to capture sub-micron resolution fluorescence images of the tissue in situ*.* The goal of this study was to quantify the in situ changes in ACL autofluorescence (AF) signal intensity and collagen microstructure as a function of the number of loading cycles.

**Methods:**

Three paired and four single cadaveric knees were subjected to a repeated 4 times bodyweight landing maneuver known to strain the ACL. The paired knees were used to compare the development of ACL microfatigue damage on the loaded knee after 100 consecutive loading cycles, relative to the contralateral unloaded control knee, through second harmonic generation (SHG) and AF imaging using confocal microscopy (CM). The four single knees were used for monitoring progressive ACL microfatigue damage development by AF imaging using CLEM.

**Results:**

The loaded knees from each pair exhibited a statistically significant increase in AF signal intensity and decrease in SHG signal intensity as compared to the contralateral control knees. Additionally, the anisotropy of the collagen fibers in the loaded knees increased as indicated by the reduced coherency coefficient. Two out of the four single knee ACLs failed during fatigue loading, and they exhibited an order of magnitude higher increase in autofluorescence intensity per loading cycle as compared to the intact knees. Of the three regions of the ACL - proximal, midsubstance and distal - the proximal region of ACL fibers exhibited the highest AF intensity change and anisotropy of fibers.

**Conclusions:**

CLEM can capture changes in ACL AF and collagen microstructures in situ during and after microfatigue damage development. Results suggest a large increase in AF may occur in the final few cycles immediately prior to or at failure, representing a greater plastic deformation of the tissue. This reinforces the argument that existing microfatigue damage can accumulate to induce bulk mechanical failure in ACL injuries. The variation in fiber organization changes in the ACL regions with application of load is consistent with the known differences in loading distribution at the ACL femoral enthesis.

**Supplementary Information:**

The online version contains supplementary material available at 10.1186/s40634-022-00507-6.

## Background

Recent cadaveric studies show that the anterior cruciate ligament (ACL) accumulates tissue fatigue damage under repetitive sub-maximal loading, and this has been hypothesized to be the mechanism underlying many of the non-contact ACL injuries, which account for about 75% of all ACL injuries [2, 4, 6, 18]. A custom three-dimensional impulsive loading device, which can simulate a cadaveric knee undergoing a single-leg jump pivot landing maneuver with realistic knee loading rates and trans-knee muscle forces (quadriceps, hamstrings and gastrocnemius), has been used to demonstrate the role of repetitive sub-maximal knee loading in causing non-contact ACL fatigue failure near the femoral enthesis, consistent with clinical observations [2, 18]. The cadaveric loading device, along with more recent in vitro ACL injury models [2], has been shown to manifest ACL injury patterns consistent with clinical observations; but these well-controlled platforms have also enabled researchers to explore parameters such as rate and type of loading, muscle forces, and underlying intra-articular stresses and strains to aid in understanding ACL injury mechanisms that would otherwise be ethically or technically challenging to study in a patient population [2, 33]. The fatigue damage signatures on the ACL generated by the sub-maximal loading device have been shown to extend across the hierarchical tissue structure, inducing structural changes of type 1 collagen at the molecular, nano (fibril), and microscale (fiber) levels. Moreover, characterization of ACL explants from patients revealed similar damage signatures, suggesting the reduction in structural integrity, in the absence of the time needed for repair, may be attributable to an overuse ACL injury [6]. A combined set of experimental and computational simulations further corroborate these findings by demonstrating that the triple-helical collagen denaturation accumulates with increasing cycles of fatigue loading due to creep strain in collagenous tissues thus leading to fatigue failure [31, 37, 38]. These studies, performed on isolated tendons in vitro, examined the progression of damage as a function of the number of cyclic loads. However, understanding fatigue damage progression is a major gap in cadaver-based ACL studies published to date [6, 33].

Recent advances in fiber optics and confocal microscopy can detect pathological signatures ex vivo and in vivo through the development of miniaturized confocal endomicroscopy with high spatial resolution that can access deeper parts of the body [15]. A benchtop confocal multiphoton microscopy (CMM) can perform second harmonic generation (SHG) imaging on tissue sections which can capture sub-micron resolution of collagen microstructures enabled by type I collagen’s intrinsic high degree of order and non-centrosymmetric structure [23]. Changes in SHG signal intensity have been used to serve as a label-free biomarker for evaluating structural integrity of tissue [27]. A confocal laser endomicroscope (CLEM) is a hand-held confocal microscope that can perform sub-micron resolution fluorescence imaging of tissues in vivo: examples include the rotator cuff tendon pathology [35], articular cartilage osteoarthritis [34], oral cancer diagnosis [8] and neurosurgery [3]. These studies involve exogenous contrast agents; however, exogenous contrast agents in vivo may not be practical due to potential immune response, cost, and challenging administration for synovial joint structures such as the ACL. On the other hand, collagen autofluorescence (AF) offers a label-free strategy to monitoring pathological conditions where alterations in tissue architecture and biochemical composition induce changes in AF [7] such as in wound healing of scar tissues [36] or inflamed arthritic mouse knees [13]. The combination of CLEM and the intrinsic fluorescence of collagen offer opportunities to expand the application toward monitoring the progression of ACL microdamage development in the knee without tissue extraction and with high spatial resolution without the need for an exogenous contrast agent.

**Fig. 1:**
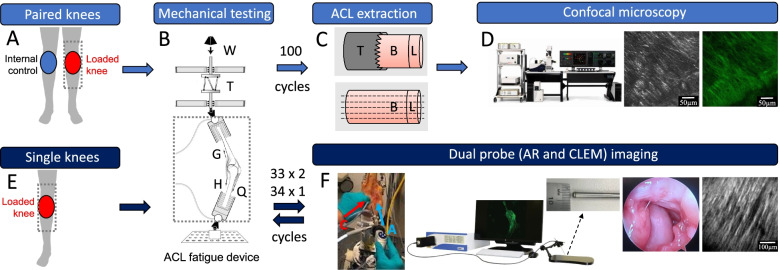
Paired knees (**A – D**) and single knees (**E – F**) experiment scheme. **A** Random selection of one of the paired knees for mechanical fatigue testing; **B** Custom in vitro loading apparatus to deliver 100 consecutive simulated sub-maximal jump landing knee loading cycles in order to repetitively strain the ACL using a weight (W) drop to apply impulsive knee compression along with an internal tibial moment via torsional device (T) activation, and quadriceps (Q), hamstring (H) and gastrocnemius (G) muscle forces. Diagram modified from Oh et al. [24]; **C** Extraction of ACL with trephine drill results in a femur bone (B) – ACL (L) explant followed by cryosection; **D** Benchtop confocal multiphoton microscopy for AF and SHG imaging of ACL sections; **E** Single knee undergoes two 33 consecutive cycles and one time 34 consecutive cycles completing a total of 100 fatigue loading cycles with interruptions due to dual probe imaging; **F** Dual probe imaging using arthroscope (blue arrow) and CLEM (red arrow) through two ports for joint visualization and AF imaging respectively. CLEM set up includes the base unit, display monitor and the 4 mm diameter handheld probe attached to the base unit

The objective of this study was to evaluate how collagen AF behaves in response to fatigue loading of the ACL and test the CLEM as a tool to capture changes in AF in ACLs in situ in the repetitively loaded knee joint. The design of the study is outlined in a simplified scheme shown in Fig. 1. In the first part of the study, we tested the hypothesis that ACL microfatigue damage will result in quantifiable changes in collagen AF signal and collagen microstructure. We used a paired knee experimental design (Fig. 1A – D) to develop microfatigue damage in one of the knees by applying 100 consecutive sub-maximal ACL fatigue loading cycles, then compared the changes in AF, and collagen microstructure between the control and fatigued knees. These measurements (AF and SHG) were taken with a bench top CMM, which required extracting the ACL from the knee and preparing the tissue to be suitable for microscopy. The second part of the study builds on the first hypothesis; we tested whether the *progressive development* of ACL microfatigue damage will result in quantifiable changes in collagen AF signal and collagen microstructure using a handheld CLEM, which enables in situ measurements. We used a single knee experimental design (Fig. 1E-F) to progressively develop microfatigue damage by repeating multiple sequences of fewer consecutive mechanical loading cycles to evaluate the changes in AF and collagen microstructure.

## Methods

### Preparation of cadaveric knees

Ten knees - three paired knees and four single knees - were acquired from the Gift of Life Anatomic Donations Program and Gift of Life Michigan (Table 1). We followed previously described procedures for preparing and mounting the adult human knees for mechanical loading [6]. The proximal femur and distal tibia were cut to a length of 20 cm from the knee joint line and mounted in cylindrical grips using polymethylmethacrylate.

**Table 1:** Demographic information for the 10 knees

	Paired knees			Single knees			
Specimen	P1	P2	P3^a^	S1	S2	S3	S4
**Sex**	M	M	F	F	F	F	F
**Age (y)**	34	36	31	38	30	38	26
**Tested limb**	L	L	R	L	R	L	R
**Height (cm)**	185	175	–	160	172	165	167
**Weight (kg)**	73	82	68	58	82	48	61
**PTS (°)**	5.2	5.9	2.0	0.9	9.0	9.1	4.8
**Total loading cycles**	100	100	100	5	9	50	105

3 paired (P) knees, of which one of each pair would serve as an unloaded control, and the 4 single (S) knees used for repeated measures experiments. PTS denotes lateral posterior tibial slope; *M* male, *F* female, *L* left, *R* right

^a^Cadaver height not available

### Paired knees for consecutive fatigue loading experiment

#### Fatigue loading

One of each pair of knees was randomly selected for fatigue loading, while the other was used as an untested control (Fig. 1A). The custom mechanical knee loading device used to fatigue the ACL [6, 18, 32, 33] is shown in Fig. 1B with a brief overview of the operation. Each knee underwent preloading cycles prior to the jump landing loading cycles to adjust the weight and distance dropped to apply a 4-times bodyweight impulsive load to the knee that was initially held in 15 degrees of flexion using trans-knee quadriceps, hamstrings and gastrocnemius muscle forces. Four times bodyweight force is reflective of the peak ground reaction force experienced during a jump landing maneuver. The mechanical loading was repeated for 100 loading cycles or until the ACL failed (> 3 mm cumulative anterior tibial translation). Details of the loading cycles are provided in the SI. The lateral posterior tibial slope (PTS) of all fatigue loaded specimens was measured as described by Hudek et al. [14] using three-dimensional T2-weighted, proton-density MRI scans acquired with a 3.0-T MRI system (Ingenia model, Philips Medical Systems; repetition time: 1000 ms; echo time: 35 ms; slice thickness: 0.7 mm; pixel spacing: 0.49 × 0.49 mm; spacing between slices: 0.35 mm; field of view: 330 × 200 × 96 mm [inferior – superior, anterior – posterior, medial – lateral, respectively]).

#### Preparation of ACL explants from paired knee cadavers

After fatigue loading was completed for one of each pair of knees, both control and loaded ACL were extracted using a trephine drill (akin to a hole saw) to preserve the femur bone and ligament insertion site. Then each fresh explant was embedded in a cryosection media to commence cryosectioning at − 25 °C. From each explant, six 20 μm thick sections were produced using the Kawamoto method, transferred on to a glass slide then preserved in − 25 °C freezer until imaging (Fig. 1C) [6].

#### SHG and AF imaging with confocal multiphoton microscopy for ACL explants

The procedures for SHG imaging were carried out using a Leica SP8 confocal multiphoton microscope (Leica Microsystems, Inc) equipped with a 910-nm Coherent Chameleon 2-photon IR laser (10% laser power, 33% gain, 38% off-set, pinhole wide open). Autofluorescence images were collected using a white light laser source (20% laser power, 50% gain) tuned to 488 nm with the same instrument. (Fig. 1D). Details of imaging are provided in the SI.

#### SHG and AF image analysis of ACL explants

The SHG and AF image intensity values were quantified with Fiji (National Institutes of Health). For each image, the mean intensity of a 100 × 100 μm area was measured for all fatigue loaded and control knees. Then the intensity values from the ACLs of tested knees were reported as a percentage of that of their contralateral control, set as the baseline intensity.

### Single knees for progressive fatigue loading experiment

#### Preparation for CLEM and single knee fatigue loading

With the knee in flexion, two small incisions were cut with a scalpel through the skin, fascia and joint capsule, one medial and one lateral to the patellar tendon for each knee. Each port formed a tunnel through which the arthroscopy (AR) and the CLEM probes could be introduced intraarticularly to visualize the joint internally and conduct the autofluorescence imaging of the ACL. The 4 mm diameter AR probe (Model 1288 HD with an X8000 Light Source, Stryker Inc., Kalamazoo, MI) was used during isotonic saline irrigation with a DePuy Duo Fluid Management System (DePuy Mitek, Inc., Raynham, MA) for guiding the 4 mm diameter CLEM probe (Optiscan, Model ‘Five2 (ViewnVivo)’, Optiscan Imaging Ltd., Mulgrave, VIC, Australia) to its region of interest on the ACL. A custom probe holder was used to stabilize the probe during image acquisition and aid in maintaining the probe normal to the surface of the ACL tissue to enable optimal image acquisition. The holder was mounted on a lockable steel gooseneck arm to maintain the desired location and orientation. CLEM and AR images were then obtained before mechanical loading of the knee from the proximal, midsubstance and distal ACL regions. The method of loading was the same as the paired knee specimens, but the loading sequence was interrupted to observe the process of microfatigue damage development. The development of any microfatigue damage in those regions was observed by capturing autofluorescence images with CLEM approximately every 33 impulsive mechanical loading cycles of the knee, until either ACL failure was observed, or 100 loading cycles was reached (Fig. 1E-F, Table 2). The cumulative internal tibial rotation (ITR) and anterior tibial translation (ATT) are reported for all specimens in Table S1.

**Table 2:** Summary of the repeated measures experimental design for each single (S) knee

Specimen	Mechanical fatigue loading and CLEM imaging sequence
**S1**	Image → 5 preloading cycles → tibial avulsion → dissect → image
**S2**	Image → 5 preloading cycles → 4 cycles (> 3-mm ATT) → image
**S3**	Image → 5 preloading cycles → image → 33 cycles → image → 45 cycles → image
**S4**	Image → 5 preloading cycles → 33 cycles → image → 66 cycles → image → 100 cycles → image

ATT denotes anterior tibial translation

#### AF imaging with CLEM for single knees

The autofluorescence images were collected with Five2 (ViewnVivo), a CLEM probe with 4 mm diameter. The light source was a 488 nm visible light laser (500 μW, 94% brightness, 2400 gain, 1x zoom 475 × 475 μm field of view, 1024 × 1024 pixels, lateral resolution: 0.55 μm, axial resolution: 5.1 μm). The detection filter was set to LP515 (longpass 515 nm). CLEM offers optical sectioning in a z-stack image sequence mode which allows image capture at various Z planes much like the CM. From each specimen, two to five locations were sampled from each of the proximal, midsubstance and distal regions of the ACL resulting in a total area of 1,353,750 μm^2^ – 3,384,375 μm^2^ across all Z planes (5 μm each) to cover the entire tissue thickness. For consistency, the selection of the three regions for all specimens was made by the orthopaedic surgeon.

#### CLEM AF image signal quantification

The brightness of the autofluorescence images was quantified using Fiji (National Institutes of Health). Fiji is an open-source image processing package that facilitates scientific image analysis. The image with the highest brightness in the Z planes was selected to represent the autofluorescence intensity from the specific loading cycle. Then the changes in AF intensity as a function of loading cycles were reported as raw mean values and cumulative percentage change in reference to the AF intensity of the ACL prior to fatigue testing, set as the baseline intensity.

#### CLEM AF image collagen fiber organization analysis

Evaluation of the changes in collagen microstructure was achieved by mapping the distribution of fiber orientations in the AF images captured during progressive fatigue loading. The Fiji plugin, OrientationJ (National Institutes of Health), was used to evaluate local orientation and anisotropic properties of each image. Prior to running the analysis, raw AF images were processed to enhance brightness and contrast for optimal fiber detection with the software (See Fig. S1 for exact steps). The output from the OrientationJ analysis, in terms of distribution and dominant direction, was the histogram of orientation angles, a coherency parameter and a color-coded map of fiber orientations. The changes in the distribution of orientation in the histogram were scrutinized rather than the absolute orientation values. The changes in fiber orientation distribution were compared by calculating the full-width-half-max (FWHM) value. A coherency coefficient was used to quantify the degree of anisotropy in fiber orientations using values ranging between 0 and 1, with 1 indicating highly oriented structures and 0 indicating anisotropic areas. For these analyses, a region of 10 pixels was designated as the local window for cubic spline interpolation.

### Statistical methods

Two-way paired t-tests were performed with Microsoft Excel™ and Python™ to test for statistically significant differences of the mean in SHG, AF signal intensity, coherency coefficient measurements between control and loaded cadaveric knees from the paired knee specimens. The significance was set at *P* ≤ 0.05. Additionally, the statistical significance of the cumulative change in AF signal intensity and coherency coefficient in single knee specimens from the final versus the initial mechanical loading sequence were performed using the same method. Then a two-way ANOVA test was used to determine the statistical significance of the changes in AF and coherency coefficient as an effect of progressive mechanical loading, or the regional differences of the ACL, or a combination of both effects. A post hoc power analysis was conducted for all the mentioned measurements using G*Power software (v. 3.1.9.7; Heinrich-Heine-Universität Düsseldorf, Düsseldorf, Germany). Details of the power analysis are provided in the SI.

## Results

### Microfatigue damage development detected by SHG and AF imaging using CMM in paired knee specimens

**Fig. 2:**
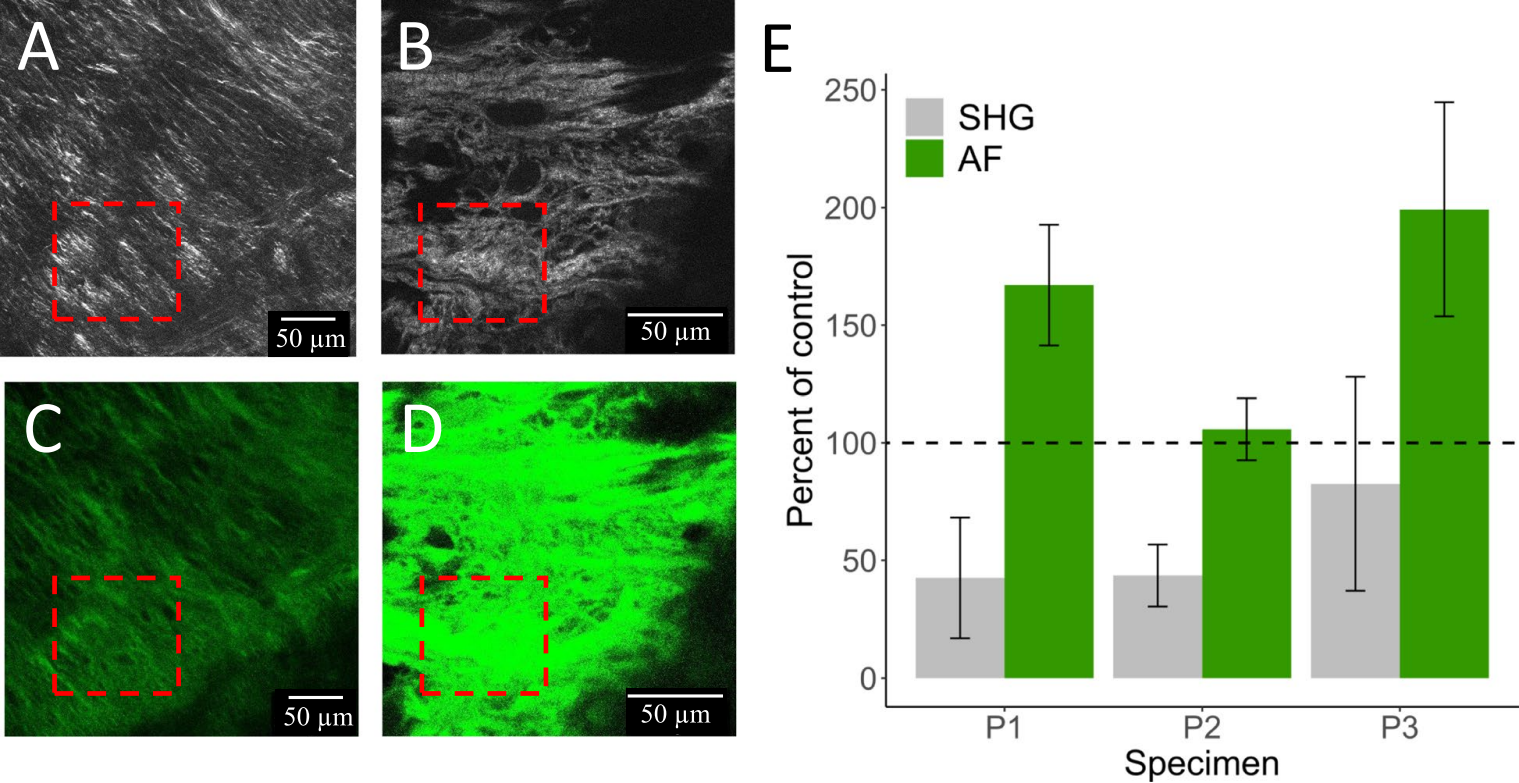
Representative images of SHG and AF images from specimen P1 using benchtop CMM and compiled paired specimen results. **A** Control ACL SHG, **B** Fatigue loaded ACL SHG, **C** Control ACL AF, **D** Fatigue loaded ACL AF, **E** The results of all paired knee samples P1 – P3 reported as the intensity of fatigue loaded ACL as a percentage of contralateral control ACL represented by the dashed line at 100%. All fatigue loaded ACLs displayed a reduced SHG signal intensity compared to their contralateral control, whereas the AF signal intensity was enhanced. Red dashed boxes (100 × 100 μm^2^) indicate regions of interest (ROI) used to quantify SHG and AF intensity

**Fig. 3:**
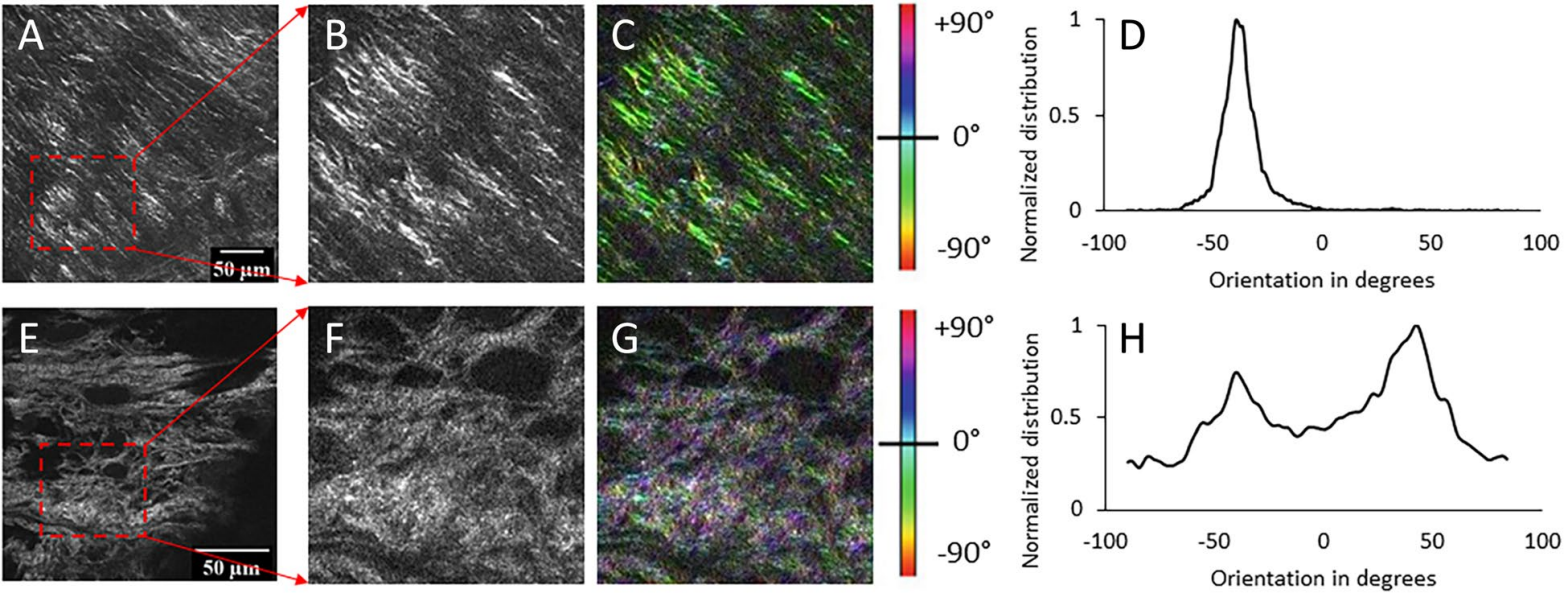
Representative fiber orientation distribution analysis results of SHG images of control (**A-C**) and fatigue loaded ACLs (**E-G**) from specimen P1. **A** SHG image of a control ACL, with the cropped ROI shown in **B**; **C** Color-coded SHG image showing orientation map of fibers; **D** Normalized distribution of orientation with a narrow peak with FWHM of 15°; **E** SHG image of a fatigue loaded ACL with the cropped ROI shown in **F**; **G** Color-coded SHG images showing orientation map of fibers; **H** Normalized distribution of orientation displays a dispersed bimodal distribution with a FWHM of 120°

**Fig. 4:**
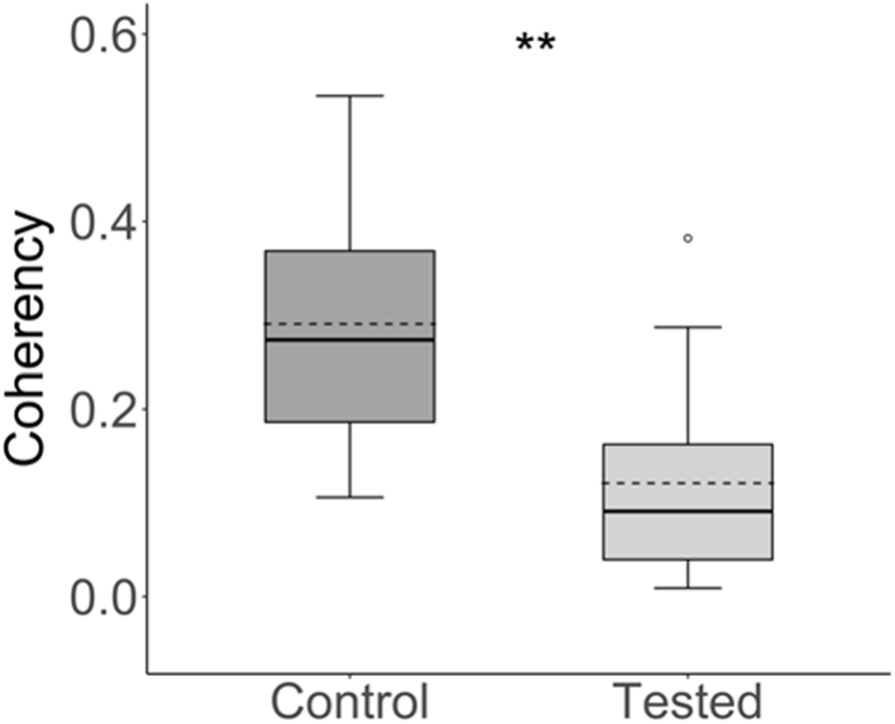
Coherency coefficient of control and fatigue loaded ACL fibers in SHG images from paired knee specimens (P1 - P3). Loss of coherency in the tested ACLs (average coherency coefficient in dashed line: 0.12. ± 0.09) compared to control ACLs (average coherency coefficient in dashed line: 0.29 ± 0.15) indicates a reduction of fiber alignment from fatigue loading (*P* = 0.003). (solid line: median, box: 1st – 3rd Quartile range, whiskers: 5 – 95% of data, dot: outliers)

Representative SHG and AF images comparing a paired control and fatigue loaded ACL from specimen P1 are shown in Fig. 2A - D. Images from specimens P2 and P3 can be found in Fig. S2. The SHG and AF signal intensity values of loaded knees are reported as a percentage of each contralateral control, set as the baseline intensity at 100% (Fig. 2E). Quantification of the paired knee specimens, P1 – P3, revealed the SHG signal intensity of fatigue loaded ACL fibers was reduced by on average 50% (*P* = 0.001), whereas the AF signal intensity increased on average by 40% (*P* = 0.02) compared to its contralateral control. Evaluation of the collagen fiber organization of the same regions in the SHG images indicated an increase in anisotropy in the fatigue loaded ACL (Fig. 3) The control ACL fiber orientation distribution showed a single peak with a full width half max (FWMH) of 15° (Fig. 3D). In contrast, the fatigue loaded ACL displayed a bimodal structure with a FWHM spanning nearly 120° (Fig. 3H). (The orientation angles are defined arbitrarily; therefore, the distribution described by FWHM is the most meaningful parameter to quantify the changes in distribution of fiber orientations.) The alignment of the fibers was represented by a coherency coefficient in all control versus fatigue loaded ACL fibers which showed the fatigue loaded ACL fibers underwent a loss of alignment (*P* < 0.005) (coherency coefficient 0.12. ± 0.09) compared to each paired control ACL (coherency coefficient 0.29 ± 0.15) (Fig. 4).

### Microfatigue damage progression as a function of fatigue loading cycles detected by AF imaging using CLEM in single knee specimens

Four single knee loading experiments were performed in which the ACL in two knees (S1, S2) failed prior to completing 100 loading cycles due to experiencing more than 3 mm cumulative ATT. Results of changes in AF and coherency values detected by CLEM as a function of fatigue loading are shown in Fig. S3. In summary (Table 3), regardless of failure status, all four specimens increased in AF intensity (*P* < 0.0001 except S3) with progressive loading cycles while the coherency was reduced (*P* > 0.05), in agreement with the results detected by the CMM. However, specimen S4 showed a slight increase in coherency. Additionally, the total change in AF intensity sustained over the course of the loading sequence was not necessarily higher in specimens with ACL failure; however, on average, the knees in which the ACL ruptured (S1, S2) had a 3.65% change in AF signal intensity per loading cycle compared to the knees in which the ACL did not rupture (S3, S4); the latter exhibited an order of magnitude lower change in autofluorescence of 0.25% increase per cycle. Surprisingly, the change in the coherency value for specimen S2 was very low, indicating little change in fiber organization, considering the ligament had failed. Evaluating these changes separated by ACL region may provide more insight. The relationship between the depth of AF image collection and the intensity of the AF signal obtained using CLEM was evaluated and determined not to have been convoluted with our analysis of the AF signal intensity (Fig. S4).

**Table 3:** Single knee ACL specimens’ overall AF and coherency changes sustained throughout the loading cycles as detected by CLEM

Specimen	AF and coherency results				
	Cumulative loading cycles ^a^	ACL status^b^	Cumulative AF change (%)	Avg. AF%/cycle	Cumulative coherency^c^ change
**S1**	5	Failed	19.6****	3.9	−0.18
**S2**	9	Failed	30.9****	3.4	−0.02
**S3**	50	Not failed	11.5	0.2	−0.23
**S4**	105	Not failed	35.8****	0.3	0.02

^a^Including pre-loading cycles

^b^Determined by cumulative ATT threshold of 3 mm sustained by ACL at the end of fatigue loading

^c^Coherency values range from − 1 (low) to 1 (high) to indicate degree of fiber alignment

^****^P < 0.0001

### Microfatigue damage progression separated by ACL region detected by AF imaging using CLEM in single knee specimens

**Fig. 5:**
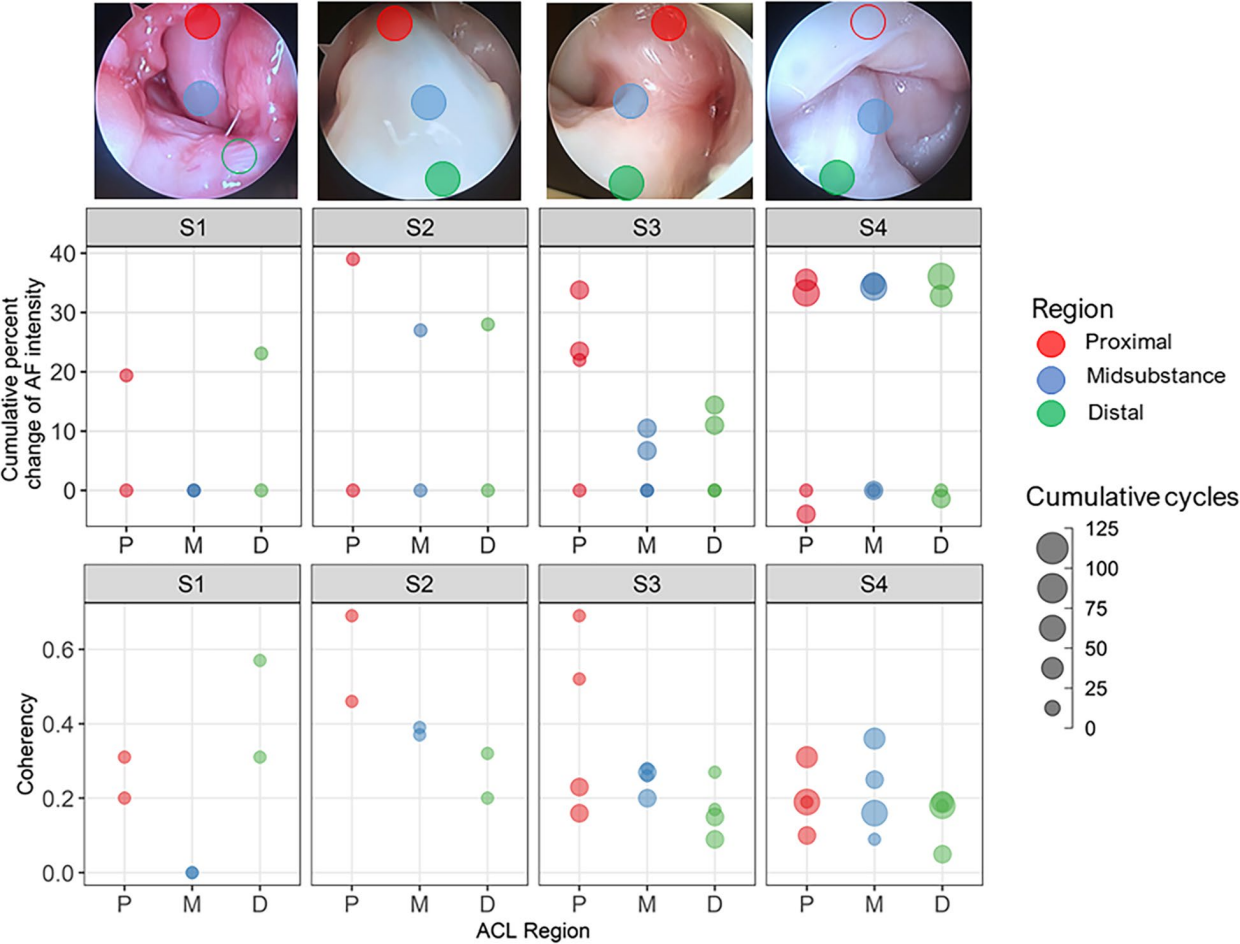
Changes in AF intensity and coherency as a function of cumulative fatigue loading cycles separated by ACL region from single knee specimens. Approximate CLEM probe placement marked by circles in the proximal (red), midsubstance (blue) and distal (green) regions in AR image (top row). Open circles indicate continuation of ACL tissue but hidden by surrounding structures. Changes in AF intensity as cumulative percentage (middle row) and coherency (bottom row) as a function of cumulative fatigue loading cycles represented by increasing circle size for every 25 loading cycles

The changes in AF intensity and the corresponding coherency values separated by ACL region (proximal, midsubstance and distal) are shown in Fig. 5 with the approximate locations of CLEM probe placement in the AR images. The optical AR and autofluorescence CLEM images of each specimen with progressive loading cycles are shown in Supplementary Figs. S5, S6, S7 and S8. Specimen S1 underwent a tibial avulsion after 5 preloading cycles, leading to a greater increase in autofluorescence signal of 23% in the distal region fibers of the ACL compared to the proximal region. The midsubstance region was unable to be imaged due to tissue damage. Specimen S2 reached failure after 5 preloading and 4 loading cycles, leading to the largest autofluorescence increase of 40% in the proximal region compared to similar changes sustained between midsubstance and distal regions with 27% and 28%, respectively. Specimen S3 possessed a thick synovial sheath; the proximal region was gently removed using a swab and the midsubstance and distal regions were cleared after 5 preloading cycles, which disintegrated the sheath. The proximal region of ACL fibers, where the synovium sheath had been cleared, displayed the largest change in AF intensity of 34% followed by the midsubstance and distal regions which sustained changes of 10% and 14%, respectively. Specimen S4 sustained the largest number of loading cycles of all single knee specimens. There was little to no change in autofluorescence in all three regions of the ACL during the first 38 cycles. However, during the 38th to the 71st cumulative loading cycles, there was an AF increase of approximately 35% for all three regions of the ACL. This value proved to be a plateau and no further increase in autofluorescence was observed over the last 34 cycles. These results show that the distribution of AF intensity change per cycle was non-linear and heterogeneous among the three regions of the ACL; this was especially nuanced in specimen S2 and S3. In particular, Specimens S2 and S3 both exhibited 10 – 20% higher AF intensity change in the proximal region compared to the other regions of the ACL.

The corresponding changes in the coherency parameter indicate that the proximal region of fibers underwent the largest loss of alignment while the midsubstance and distal region fibers were affected to a lesser degree (Fig. 5). For specimen S1, the largest change in coherency was seen in the distal region, in agreement with the largest AF signal change and tibial avulsion. Specimen S4 displayed a uniform magnitude of change in AF and coherency among the three regions throughout the loading sequence, showcasing that an equilibrium response to loading from all three regions of the ACL leads to the least disruption in fiber organization compared to the other single knee specimens. Results of the ANOVA test indicate that the changes in AF and coherency coefficient in all the single knee specimens are statistically significant in response to either the effect of fatigue loading (*P* ≤ 0.001) or the ACL regional variation (*P* ≤ 0.05) or a combination of both effects. Previous work indicates that dehydration of tissue can also result in disruption of collagen triple-helix structure [6]. As a precautionary measure, we performed a separate control experiment to explore dehydrated-related collagen unraveling as the driver of changes in autofluorescence (Fig. S9). The results showed that the dehydration-based changes did not generate the magnitude of autofluorescence observed from the structural changes induced by the repetitive pivot-landing loading regimen.

## Discussion

We present the first study to employ a commercially available CLEM to capture the development of molecular level microfatigue damage and visualize fiber-level collagen organization changes in human ACLs using AF signal alone. The CLEM allowed the ACL to be preserved inside the knee during repetitive mechanical loading inducing knee flexion and internal tibial rotation in a realistic scenario of how ACL strain develops in the knee. Our results confirm that using CLEM, the AF signal and the fiber-level collagen structures can be measured and quantified to demonstrate the progression of microfatigue damage accumulation in ACLs. The findings give new insight into the physical mechanisms underlying changes in AF signal in response to microfatigue damage, AF trends and regional variation of the ACL microfatigue damage accumulation process.

The ACL owes its mechanical strength to its hierarchical architecture of its primary structural protein, type 1 collagen [22, 25, 30]. Type 1 collagen’s unique high degree of order and non-centrosymmetric structure and its chemical constituents makes the material ideal for the application of label-free imaging modalities which can serve as structural probes, such as SHG and AF imaging [7, 16]. A loss of SHG signal indicates a disruption of collagen hierarchical organization [6, 16], therefore, an average of 50% of reduction in SHG signal with a 40% increase in AF after 100 sub-maximal consecutive loading cycles in the paired knees (Fig. 2) indicates: i) a disruption of collagen’s hierarchical organization as a likely physical mechanism for changes in AF signal, and ii) AF as a potential tool that is sensitive enough to detect multi-scalar microfatigue damage complementary to SHG imaging. The inverse SHG and AF signal development is consistent with previous studies reporting an unraveling of the collagen molecule due to destabilization of the hydrogen bond network in various collagen-based materials [6, 16, 17]. AF can capture information such as the tissue’s molecular environment that depend not only on the endogenous fluorophores but also on their architecture due to its effect on optical properties of tissue such as absorption or scattering of light [12]. Collagen contains fluorophores such as phenylalanine, tyrosine, pyridinoline cross-links and the advanced glycation end products where about 50% of these fluorophores are located within a cross-link site [10, 20, 28]. Supported by the reduction in SHG signal intensity and the coherency coefficient (Figs. 3 and 4), the physical mechanism of the increase in AF can be attributed to molecular level structural damage causing the fluorophores around the cross-link site to separate from one another, leading to reduced fluorescence quenching. For example, tyrosine residues, which are typically wrapped in the non-helical telopeptide regions near the cross-links, increase tissue autofluorescence when type 1 collagen is dissociated due to the increased number of exposed tyrosine residues [28]. This unravelling process disturbs the hydrophobic interactions the residues provide, which aids in the collagen self-assembly process [26]. Interestingly, the average increase in AF intensity per loading cycle in the specimens with failed ACL (S1, S2) exhibit at least an order of magnitude larger amount than specimens S3 and S4 which did not fail (3.65% per cycle vs 0.25% per cycle, respectively). This suggests a larger AF signal intensity change occurs when the ACL is nearing the end of its fatigue life. However, the real distribution per cycle in the failed specimens S1, S2 are unknown due to the lack of AF measurements between the initial and final point of the fatigue loading sequence. A finer sampling measure during the fatigue loading cycles will be needed to answer exactly how the AF behaves near the end of the ACL fatigue life.

The ACL is known to manifest inhomogeneous strain especially near the bone-enthesis-ligament region due to the angle of attachment as well as fiber splay, which engages some fibers more than others [19, 21]. This is consistent with the common site of non-contact ACL complete ruptures near its femoral enthesis [18, 33]. Figure 5 shows changes in AF and coherency of single knee specimens separated by three regions of the ACL - proximal, midsubstance and distal. The evaluation combines measurements taken from both the anteromedial (AM) and posterolateral (PL) bundles as they were not identified in this study; however, Skelley et al. reported that the mechanical and microstructural parameters do not vary discretely by bundle but rather more gradually across the full span of the ligament [29]. Inspection of the change in AF by ACL region in Specimen S2 and S3 agrees with Skelley et al.’s observation, exhibiting a 10 – 20% higher increase in AF in the proximal region compared to the midsubstance and distal regions. Additionally, the AF measurements from Specimen S3 and S4 depict a more elaborate change in AF during progressive mechanical loading due to the finer sampling of AF measurements compared to specimen S1 and S2. This reveals a non-linear increase in AF intensity with cumulative loading cycles prior to ligament failure. Previous studies have documented the nonlinear mechanical behavior (e.g. stiffness and tensile modulus) of single tropocollagen molecule [11] and collagen fibrils [1] based on time dependence (i.e. deformation rate). It is also reported that the deformation behavior of collagen is governed by collagen’s structural hierarchy [25]. These results suggest the increase in AF reflects a collective non-linear mechanical behavior present in the collagen hierarchies and supports the possibility that a large increase in AF may occur in the final few cycles immediately prior to or at failure, representing a greater plastic deformation of the tissue. Similarly, the corresponding proximal region fibers show the highest coherency values and the largest reduction in fiber alignment, reflecting highly aligned fibers under increasing load to undergo major fiber reorganization due to stress-relaxation and or permanent damage (Fig. 5). This non-uniform structural response is consistent with the known gradient of axial displacement and loading force of the ACL under uniaxial tension [19, 21, 29], which stem from material heterogeneity across different hierarchies including fibril length, width and density [5]. A simple cyclic loading experiment under uniaxial tension demonstrated the same trend of proximal region AF changes, with the most intense signal produced at the end of the testing sequence when a partial tear was generated (Table S2 and Fig. S10). Our results are consistent with the study that showed a correlation of AF signal with the breaking force during tensile tests of connective tissues [9], leading us to suspect that the magnified growth of AF signal in the proximal region of the ACL compared to the midsubstance and distal regions indicate an impending tissue fatigue failure near its femoral enthesis. We believe the findings of this study aid in understanding the progressive changes in the tissue prior to failure and emphasize the importance of frequency and duration of loading cycles in determining ACL’s fatigue life [18], which can be utilized to inform injury prevention programs (e.g. training and conditioning regimens).

We found that robust signal detection can be hindered by poor probe-to-sample contact and any movement during image collection. Therefore, a custom probe holder was built which improved probe stability and sample contact while imaging. In addition, there was considerable biological heterogeneity in the thickness and extent of the synovial sheath covering the ACL and, when present, this limited the detection of autofluorescence signals from the ACL collagen fibers under the sheath. Therefore, a swab was used to gently brush the synovium aside without perturbing the underlying fibers. Another limitation in this initial study is the limited number of samples. This experiment employed a controlled pivot landing model on cadaver knees and cannot represent the full range of loads and mechanical challenges found in living individuals. A larger sample set including diverse age groups and morphology of the knee such as tibial slope and alpha angle would provide a more comprehensive view of how and where the AF signal develops as a function of fatigue loading cycles. Finally, CLEM can only image the surface layers of the ACL so microfatigue damage in deeper layers was unable to be evaluated.

## Conclusions

This study revealed that, during and after repetitive sub-maximal loading, CLEM can quantify changes in ACL autofluorescence and collagen microstructures measured in situ. Results suggest a large increase in AF may occur in the final few cycles immediately prior to or at ACL failure, representing a greater plastic deformation to the tissue. This reinforces the argument that existing microfatigue damage can accumulate to induce bulk mechanical failure in ACL injuries. The variation in fiber organization changes in the ACL regions with application of load is consistent with the known differences in the distribution of loading forces near the ACL femoral enthesis.

## Supplementary Information

**Additional file 1: Table S1.** Peak cumulative kinematic measures for each knee. **Figure S1.** Imaging processing steps of CLEM AF images for fiber orientation distribution and coherency analysis with Fiji and representative results. Left: ImageJ macro code for image processing steps. Right: vector field map shows orientation of vectors aligned with outlines of fibers where the longer length indicates higher coherency. Orientation map shows fiber orientation indicated by color legend (Scale bar: 100 μm). **Figure S2.** SHG and AF images of control and tested (100 cycles) paired knee specimens P2 and P3 using a benchtop CMM. Red dashed boxes (100 × 100 μm^2^) indicate regions of interest (ROI) used to quantify SHG and AF intensity. **Figure S3.** Changes in AF intensity and coherency as a function of total cycles in single knee specimens. a) Specimen S1, b) Specimen S2, c) Specimen S3, and d) Specimen S4. The AF intensity increases with increasing loading cycles while the coherency decreases, except for specimen S4. The color of circles indicates the progression of loading cycles, where the darker blue indicates higher total cycles. **Figure S4.** Single knee specimen changes in AF intensity and depth of image acquired as a function of total cycles using CLEM. a) Specimen S1, b) Specimen S2, c) Specimen S3, and d) Specimen S4. No significant trend is shown. Total cycles are normalized to indicate the end of the cycle as 1. **Figure S5.** Specimen S1 AR and CLEM AF image of ACL before load (a, c, e) and after 5 pre-load (b, d, f). Before load a) AR image showing CLEM probe placement for AF image capture at distal (green; c, d) and proximal (blue; e, f) regions of ACL. Midsubstance region was unable to be imaged due to tissue damage. Images brightness and contrast are adjusted for better visualization. Scalebar 100 μm. **Figure S6.** Specimen S2 AR and CLEM AF image of ACL before load (a, c, e, g) and after 5 pre-load and 4 cycles (b, d, f, h). Before load a) AR image showing CLEM probe placement for AF image capture at distal (green; c, d), midsubstance (red; e, f) and proximal (blue; g, h) regions of ACL. Images brightness and contrast are adjusted for better visualization. Scalebar 100 μm. **Figure S7.** Specimen S3 AR and CLEM AF image of ACL before load (a, c, g, k), after 5 pre-load (d, h, l), additional 33 cycles (e, i, m) and up to 45 cycles (b, f, j, n). Before load a) AR image showing CLEM probe placement for AF image capture at distal (green; c, d, e, f), midsubstance (red; g, h, i, j) and proximal (blue; k. l, m, n) regions of ACL. Images brightness and contrast are adjusted for better visualization. Scalebar 100 μm. **Figure S8.** Specimen S4 AR and CLEM AF image of ACL before load (a, c, g, k), after 5 pre-load and additional 33 cycles (d, h, l), up to 66 cycles (e, i, m) and up to 100 cycles (b, f, j, n). Before load a) AR image showing CLEM probe placement for AF image capture at distal (green; c, d, e, f), midsubstance (red; g, h, i, j) and proximal (blue; k. l, m, n) regions of ACL. Images brightness and contrast are adjusted for better visualization. Scalebar 100 μm. **Figure S9.** Effect of dehydration on tissue AF measured with CLEM and AFM-IR. AF images of ACL in the a) initial hydrated state b) dehydrated state subjected to N_2_ gas for 8 minutes c) rehydrated state with water d) post storage in 4 °C refrigerator for 24 hours. e) AF reported as percentage of initial state measurement remains steady from steps a – c however, increases 16% after preservation in the refrigerator for 24 hours. f) AFM-IR spectrum parallels the lack of effect of dehydration seen by a steady 1672 cm^− 1^/1740 cm^− 1^ ratio until the tissue is kept in the refrigerator which completely reduces the 1672 cm^− 1^ signal, leaving only the 1740 cm^− 1^ peak (red spectrum). **Table S2.** Tensile testing sequence parameters for femur – ACL – tibia complex (FATC) cadaveric ACL. **Figure S10.** Femur – ACL – Tibia complex (FATC) cyclic tensile test of ACL following sequence in Table S2. a) Cadaver knee with 45° flexion angle in the tensile test device. Femur was exercised up and down (yellow arrow) b) ACL with a partial tear (red dotted circle) at the proximal region during the 11th cycle of the 8th testing sequence with 200 N cyclic load at 20 mm/min. The three regions of AF image acquisition are proximal (P), midsubstance (M) and distal (D). c) The change in mean AF intensity of the image for the three regions exhibit the largest AF signal in the proximal region at the end of the testing sequence, corresponding to the hole. (standard error for each data point range was 20 – 35 not shown). d) CLEM AF images of ACLs during testing sequence number 2, 4, 6 and 8 (point of tear). Scale bar: 100 μm.

## Data Availability

The datasets used and/or analyzed during the current study are available from the corresponding author on reasonable request.

## Abbreviations

ACL: Anterior cruciate ligament
AF: Autofluorescence
AM: Anteromedial
AR: Arthroscope
ATT: Anterior tibial translation
CLEM: Confocal laser endomicroscopy
CMM: Confocal multiphoton microscopy
FWHM: Full-width half max
ITR: Internal tibial rotation
PL: Posterolateral
PTS: Posterior tibial slope
SHG: Second harmonic generation
