# Supplementary material for: Anterior cruciate ligament microfatigue damage detected by collagen autofluorescence in situ
Source: J Exp Orthop. 2022 Jul 30;9:74. doi: 10.1186/s40634-022-00507-6 (PMC9339057; doi:10.1186/s40634-022-00507-6)
Supplement: Supplementary file 1 — Additional file 1: Table S1. Peak cumulative kinematic measures for each knee. Figure S1. Imaging processing steps of CLEM AF images for fiber orientation distribution and coherency analysis with Fiji and representative results. Left: ImageJ macro code for image processing steps. Right: vector field map shows orientation of vectors aligned with outlines of fibers where the longer length indicates higher coherency. Orientation map shows fiber orientation indicated by color legend (Scale bar: 100 μm). Figure S2. SHG and AF images of control and tested (100 cycles) paired knee specimens P2 and P3 using a benchtop CMM. Red dashed boxes (100 × 100 μm2) indicate regions of interest (ROI) used to quantify SHG and AF intensity. Figure S3. Changes in AF intensity and coherency as a function of total cycles in single knee specimens. a) Specimen S1, b) Specimen S2, c) Specimen S3, and d) Specimen S4. The AF intensity increases with increasing loading cycles while the coherency decreases, except for specimen S4. The color of circles indicates the progression of loading cycles, where the darker blue indicates higher total cycles. Figure S4. Single knee specimen changes in AF intensity and depth of image acquired as a function of total cycles using CLEM. a) Specimen S1, b) Specimen S2, c) Specimen S3, and d) Specimen S4. No significant trend is shown. Total cycles are normalized to indicate the end of the cycle as 1. Figure S5. Specimen S1 AR and CLEM AF image of ACL before load (a, c, e) and after 5 pre-load (b, d, f). Before load a) AR image showing CLEM probe placement for AF image capture at distal (green; c, d) and proximal (blue; e, f) regions of ACL. Midsubstance region was unable to be imaged due to tissue damage. Images brightness and contrast are adjusted for better visualization. Scalebar 100 μm. Figure S6. Specimen S2 AR and CLEM AF image of ACL before load (a, c, e, g) and after 5 pre-load and 4 cycles (b, d, f, h). Before load a) AR image showing CLEM probe placemen [file 40634_2022_507_MOESM1_ESM.docx]

**Supplementary information for**

**Anterior Cruciate Ligament Microfatigue Damage Detected by Collagen Autofluorescence in situ**

**Methods**

*Mechanical pre-loading and fatigue loading cycles description*

For the pre-loading cycles, the torsional device was locked to prevent internal tibial torque from contributing to ACL strain. Then, the torsional device was unlocked so that for each simulated jump landing loading cycle, the dropped weight induced simultaneous impulsive 4 times bodyweight knee compression, flexion and internal tibial rotation moments peaking in ~70 ms [5] The six-axis load cells located at the distal tibia and proximal femur measured the knee 3-D input and output forces and moments.

*Confocal multiphoton microscopy imaging parameters*

Two photomultiplier detectors were set at 440 nm for the detection of the SHG signal forward and backward propagation signals. A total area of 1500 x 900 µm (1,350,000 µm^2^) was captured per tissue section by stitching 15 300 x 300 µm images across all Z planes (8.6 µm each) to cover the entire tissue thickness. The photomultiplier detector was set at 520 – 560 nm for autofluorescence detection. Autofluorescence images were captured over the same regions as measured for SHG imaging

*Post hoc power analysis results*

A two-way t-test of the SHG, AF and coherency coefficient differences from the paired knee specimens, possessed a power of 0.96, 0.98, 0.78 respectively given the significance criterion (α = 0.05), sample size (n = 6; three control ACL’s and three fatigue loaded ACL’s) and effect size ( > 1), which indicates a strong to moderate effect in the population. The same post hoc power analysis for the single knee specimens’ change in AF intensity comparing the final and initial load exhibited a power of 1 given the high effect size (es = 8.1), significance criterion (α = 0.05) and sample size (n = 4).

**Table S1.** **Peak cumulative kinematic measures for each knee.**

| **Specimen** | **ITR (°)** | **ATT (mm)** |
| --- | --- | --- |
| P1 | 0.2 | 0.1 |
| P2 | 5.3 | 6.9 |
| P3 | 9.5 | 8.5 |
| S1* | - | - |
| S2 | 1.2 | 3.0 |
| S3 | 1.9 | 1.1 |
| S4 | 0.8 | 0.1 |

ITR and ATT recorded after the last fatigue loading cycle corresponding to sample. ITR, internal tibial rotation; ATT, anterior tibial translation. ^*^ITR and ATT measurements were not available due to ligament failure during knee pre-loading conditioning cycles.

**
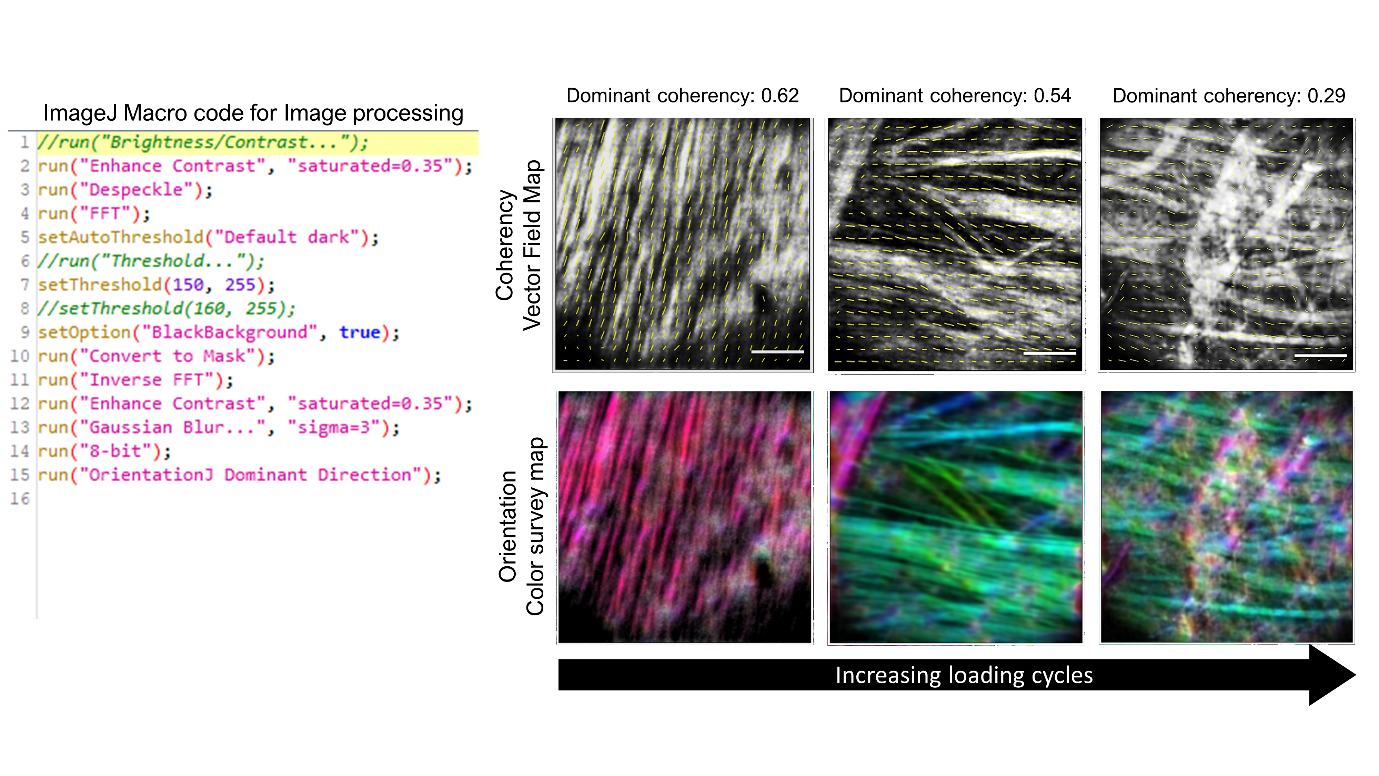
**

**Figure S1**. **Imaging processing steps of CLEM AF images for fiber orientation distribution and coherency analysis with Fiji and representative results**. Left: ImageJ macro code for image processing steps. Right: vector field map shows orientation of vectors aligned with outlines of fibers where the longer length indicates higher coherency. Orientation map shows fiber orientation indicated by color legend (Scale bar: 100 µm).


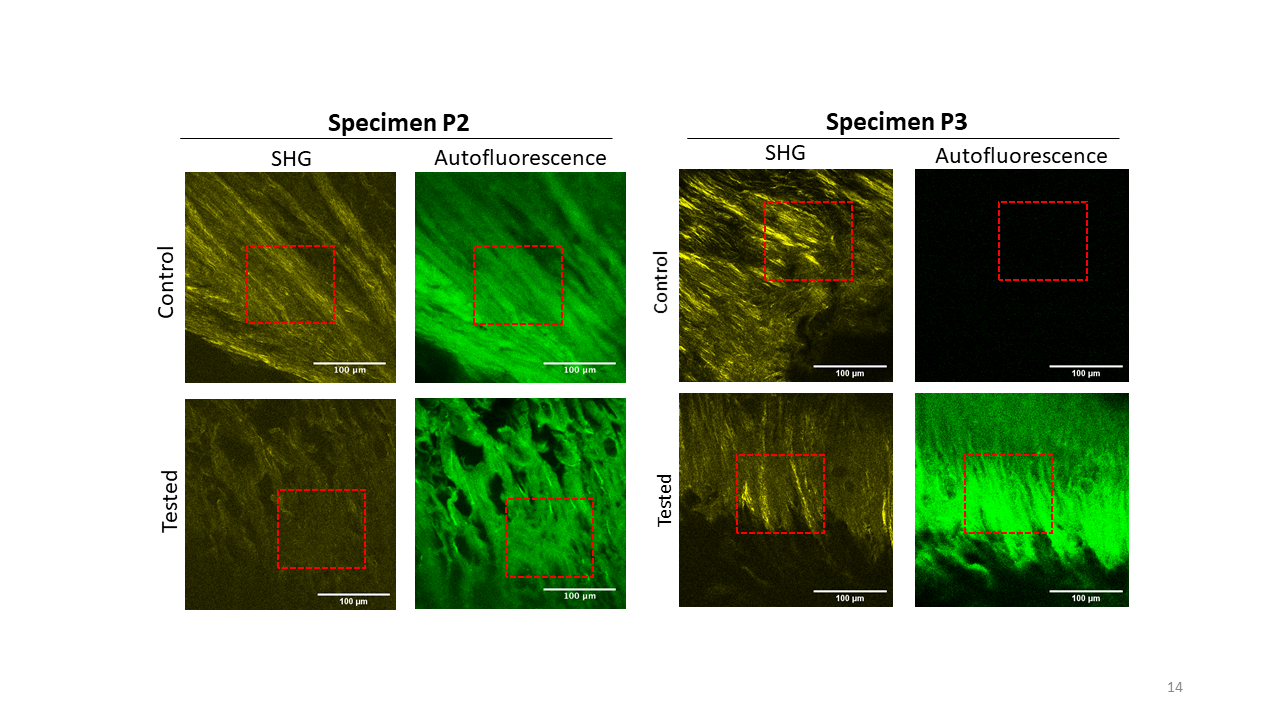


**Figure S2**. **SHG and AF images of control and tested (100 cycles) paired knee specimens P2 and P3 using a benchtop CMM**. Red dashed boxes (100 x 100 µm^2^) indicate regions of interest (ROI) used to quantify SHG and AF intensity.


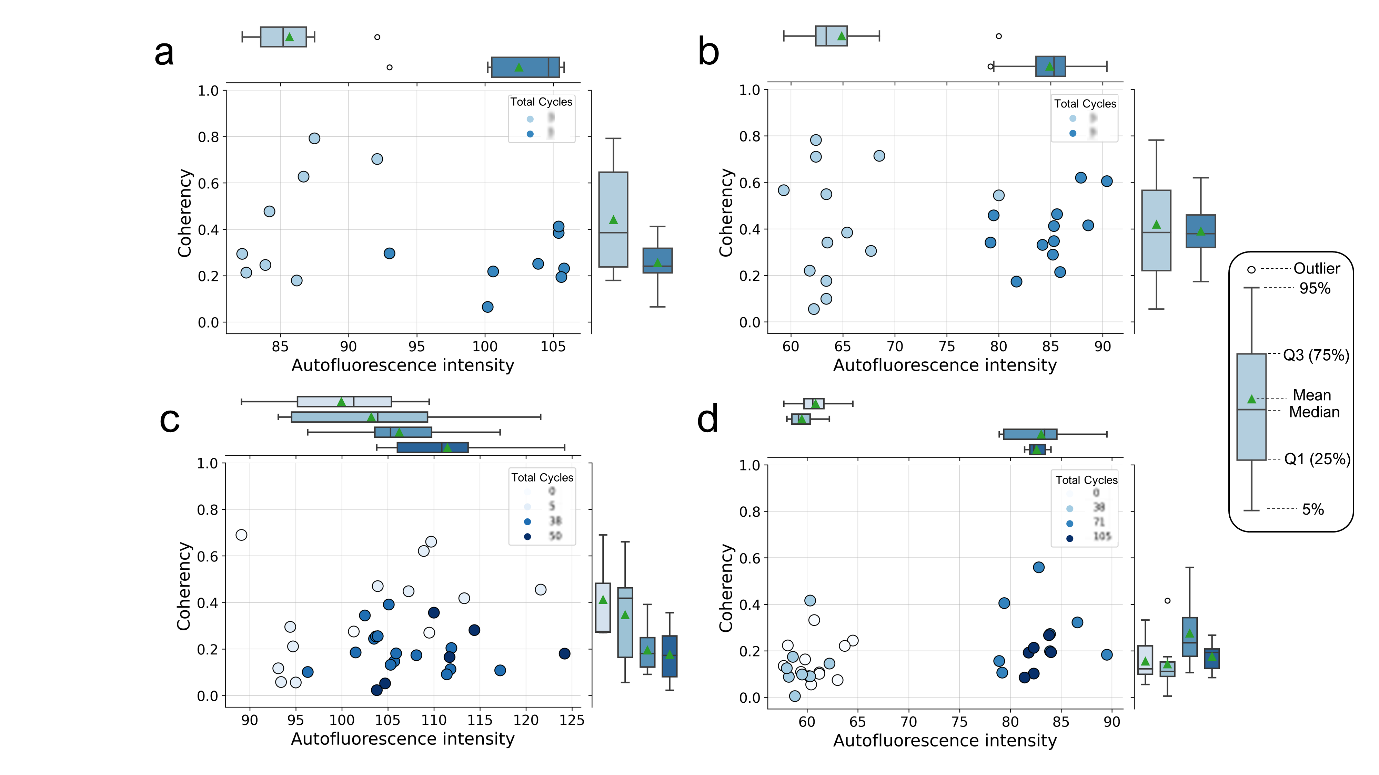


**Figure S3. Changes in AF intensity and coherency as a function of total cycles in single knee specimens.**

a) Specimen S1, b) Specimen S2, c) Specimen S3, and d) Specimen S4. The AF intensity increases with increasing loading cycles while the coherency decreases, except for specimen S4. The color of circles indicates the progression of loading cycles, where the darker blue indicates higher total cycles.


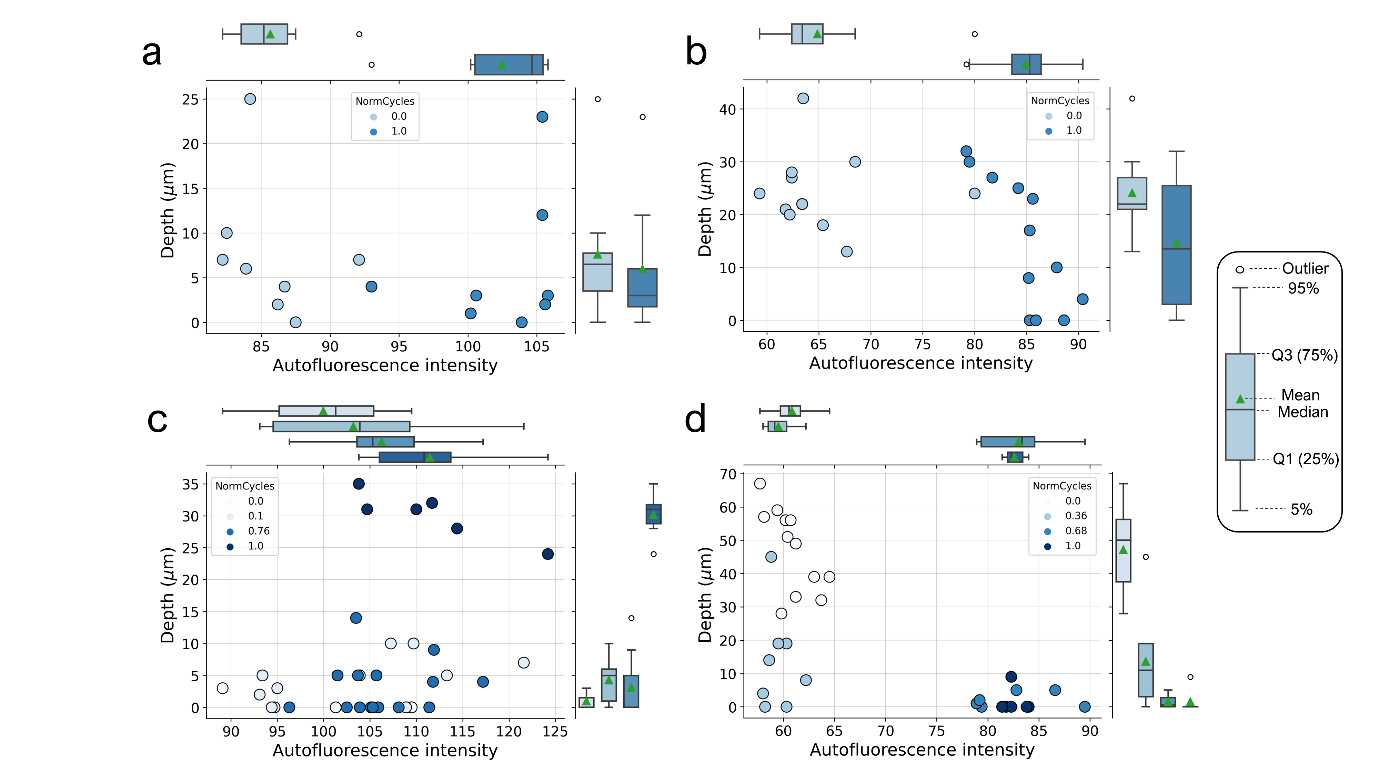


**Figure S4. Single knee specimen changes in AF intensity and depth of image acquired as a function of total cycles using CLEM.** a) Specimen S1, b) Specimen S2, c) Specimen S3, and d) Specimen S4. No significant trend is shown. Total cycles are normalized to indicate the end of the cycle as 1.


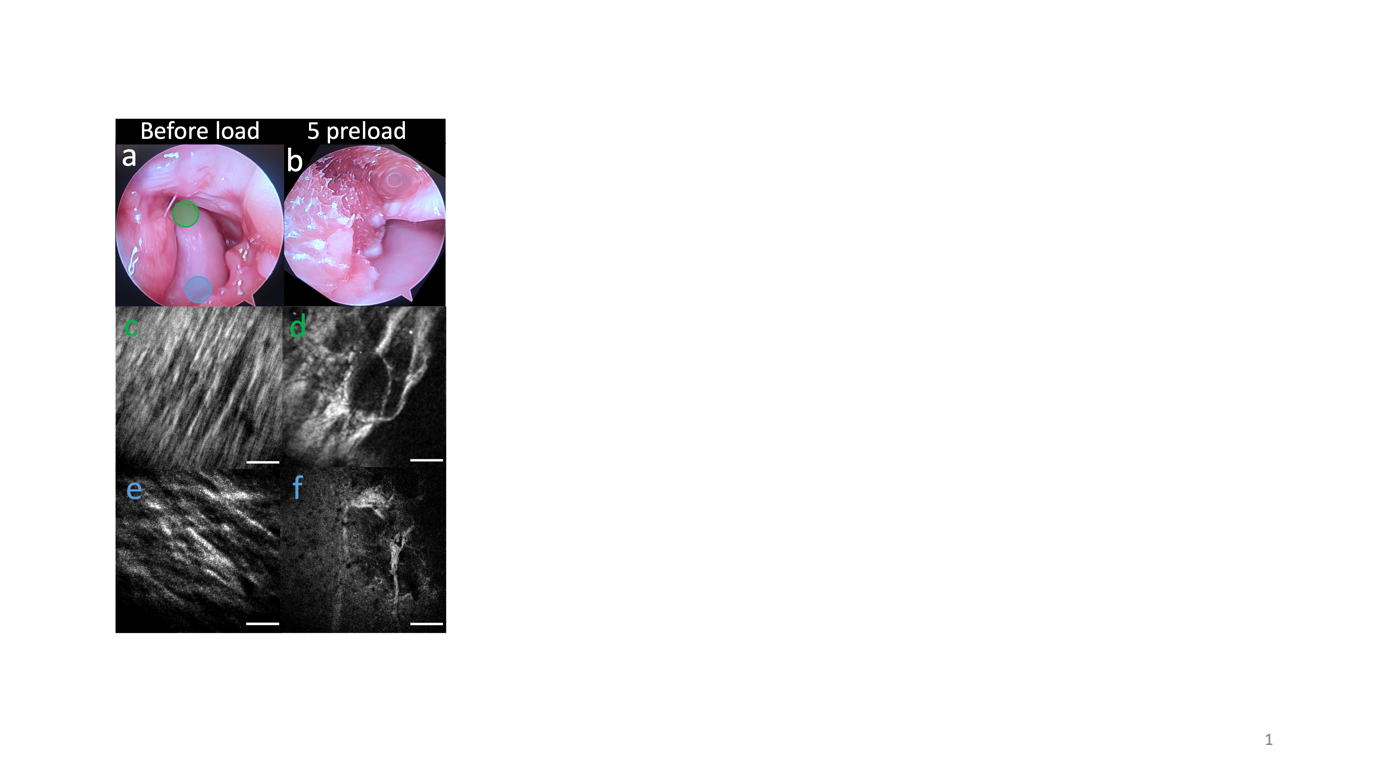


**Figure S5**. **Specimen S1 AR and CLEM AF image of ACL before load (a, c, e) and after 5 pre-load (b, d, f).** Before load a) AR image showing CLEM probe placement for AF image capture at distal (green; c, d) and proximal (blue; e, f) regions of ACL. Midsubstance region was unable to be imaged due to tissue damage. Images brightness and contrast are adjusted for better visualization. Scalebar 100 µm.


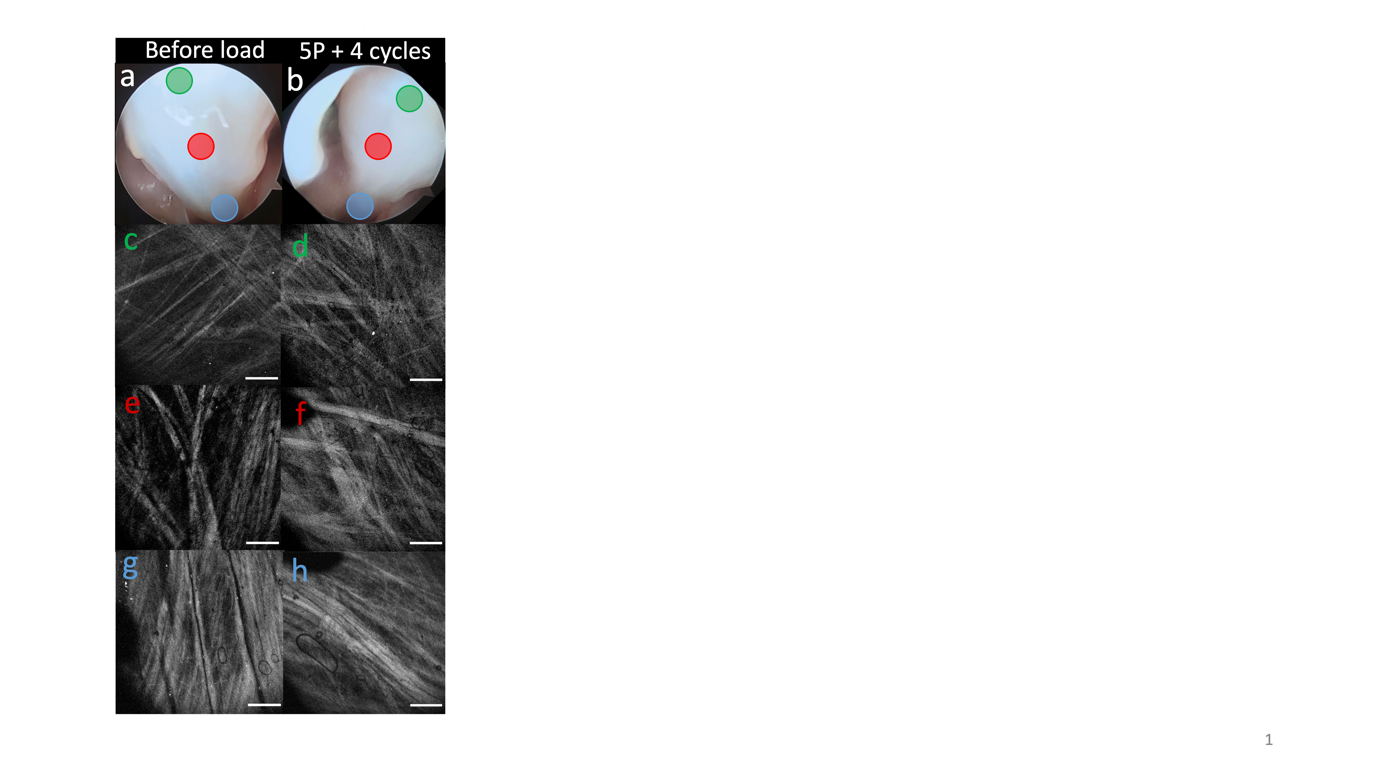


**Figure S6**. **Specimen S2 AR and CLEM AF image of ACL before load (a, c, e, g) and after 5 pre-load and 4 cycles (b, d, f, h).** Before load a) AR image showing CLEM probe placement for AF image capture at distal (green; c, d), midsubstance (red; e, f) and proximal (blue; g, h) regions of ACL. Images brightness and contrast are adjusted for better visualization. Scalebar 100 µm.


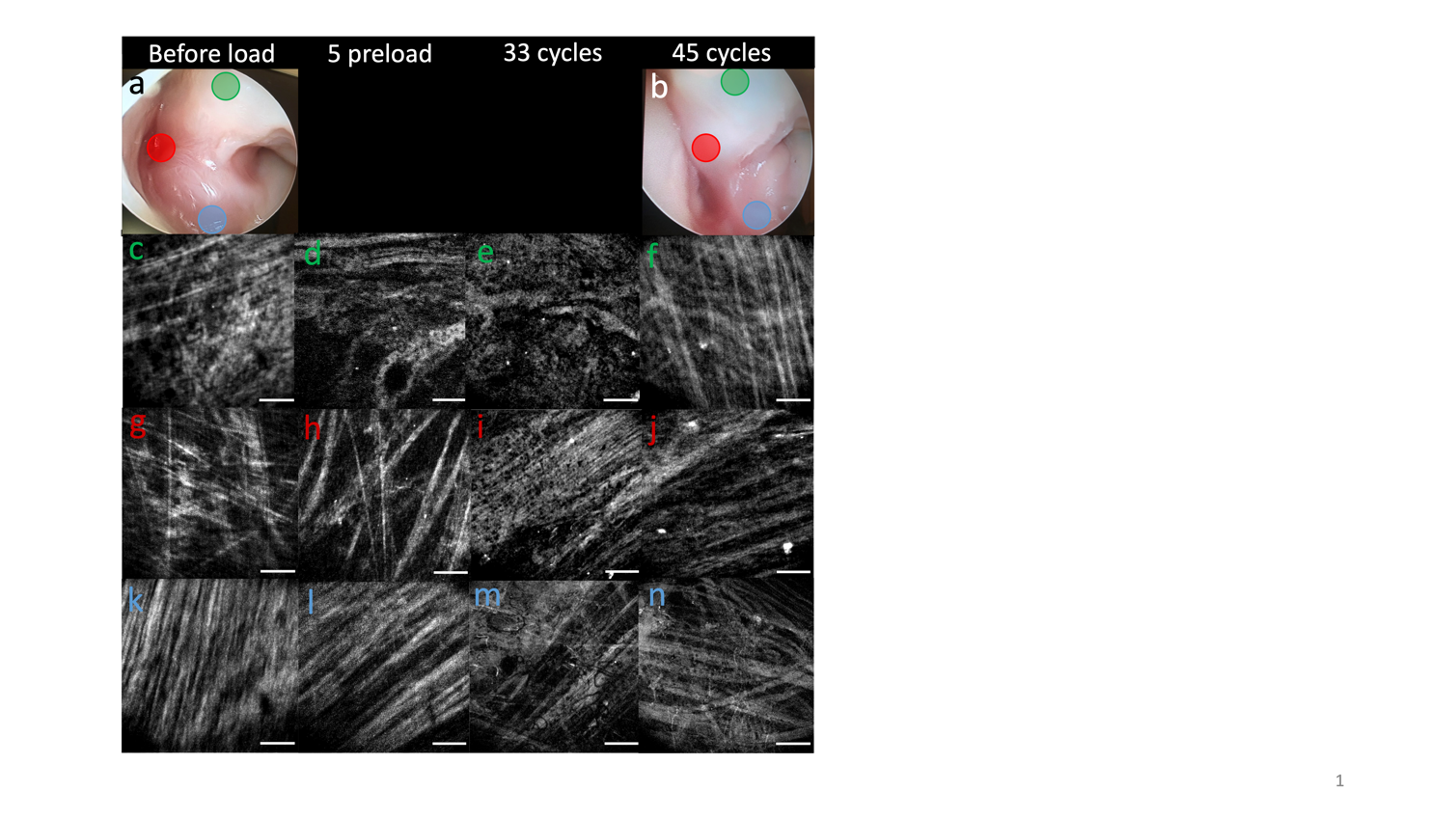


**Figure S7**. **Specimen S3 AR and CLEM AF image of ACL before load (a, c, g, k), after 5 pre-load (d, h, l), additional 33 cycles (e, i, m) and up to 45 cycles (b, f, j, n).** Before load a) AR image showing CLEM probe placement for AF image capture at distal (green; c, d, e, f), midsubstance (red; g, h, i, j) and proximal (blue; k. l, m, n) regions of ACL. Images brightness and contrast are adjusted for better visualization. Scalebar 100 µm.


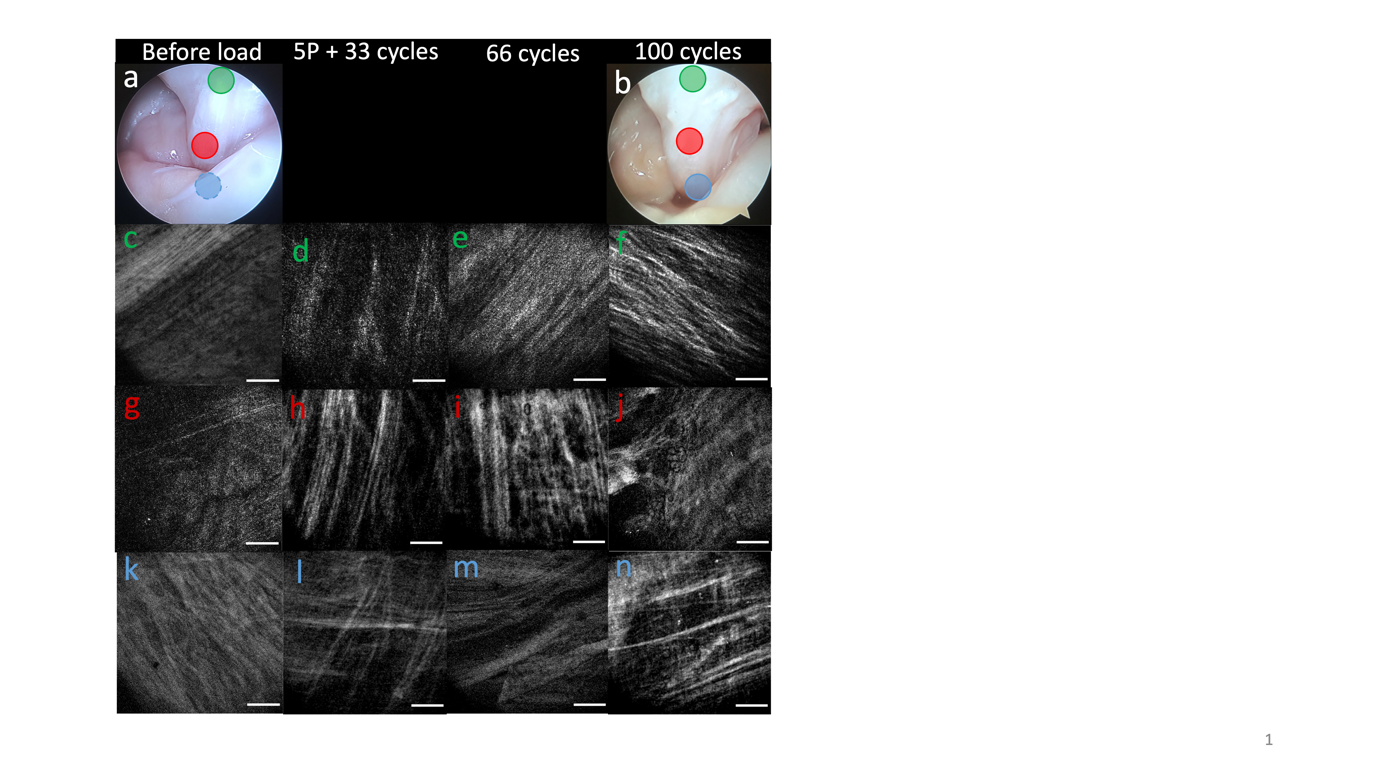


**Figure S8**. **Specimen S4 AR and CLEM AF image of ACL before load (a, c, g, k), after 5 pre-load and additional 33 cycles (d, h, l), up to 66 cycles (e, i, m) and up to 100 cycles (b, f, j, n).** Before load a) AR image showing CLEM probe placement for AF image capture at distal (green; c, d, e, f), midsubstance (red; g, h, i, j) and proximal (blue; k. l, m, n) regions of ACL. Images brightness and contrast are adjusted for better visualization. Scalebar 100 µm.


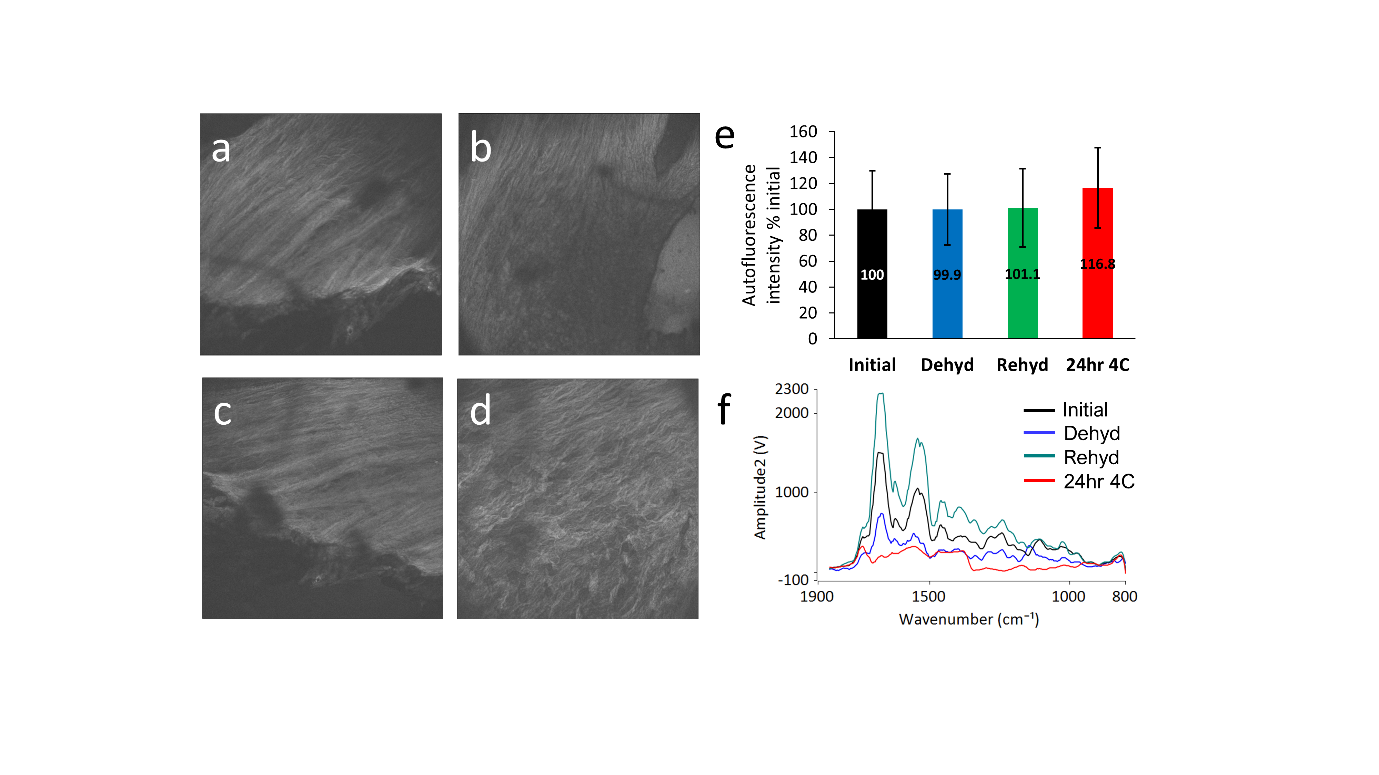


**Figure S9**. **Effect of dehydration on tissue AF measured with CLEM and AFM-IR**. AF images of ACL in the a) initial hydrated state b) dehydrated state subjected to N_2_ gas for 8 minutes c) rehydrated state with water d) post storage in 4 °C refrigerator for 24 hours. e) AF reported as percentage of initial state measurement remains steady from steps a – c however, increases 16% after preservation in the refrigerator for 24 hours. f) AFM-IR spectrum parallels the lack of effect of dehydration seen by a steady 1672 cm^-1^/1740 cm^-1^ ratio until the tissue is kept in the refrigerator which completely reduces the 1672 cm^-1^ signal, leaving only the 1740 cm^-1^ peak (red spectrum).

**Table S2.** **Tensile testing sequence parameters for femur – ACL – tibia complex (FATC) cadaveric ACL**

| **Sequence number** | **Cyclic load (N), rate, Number of cycles** |
| --- | --- |
| 1 | 2.5 N Preload, 20 mm/min for 10 cycles, 2 mm displacement |
| 2 | 50 N, 20 mm/min for 20 cycles |
| 3 | 75 N, 20 mm/min for 20 cycles |
| 4 | 100 N, 20 mm/min for 20 cycles |
| 5 | 125 N, 20 mm/min for 20 cycles |
| 6 | 150 N, 20 mm/min for 20 cycles |
| 7 | 175 N, 20 mm/min for 20 cycles |
| 8 | 200 N, 20 mm/min for 11 cycles – ACL tear at proximal region |

**
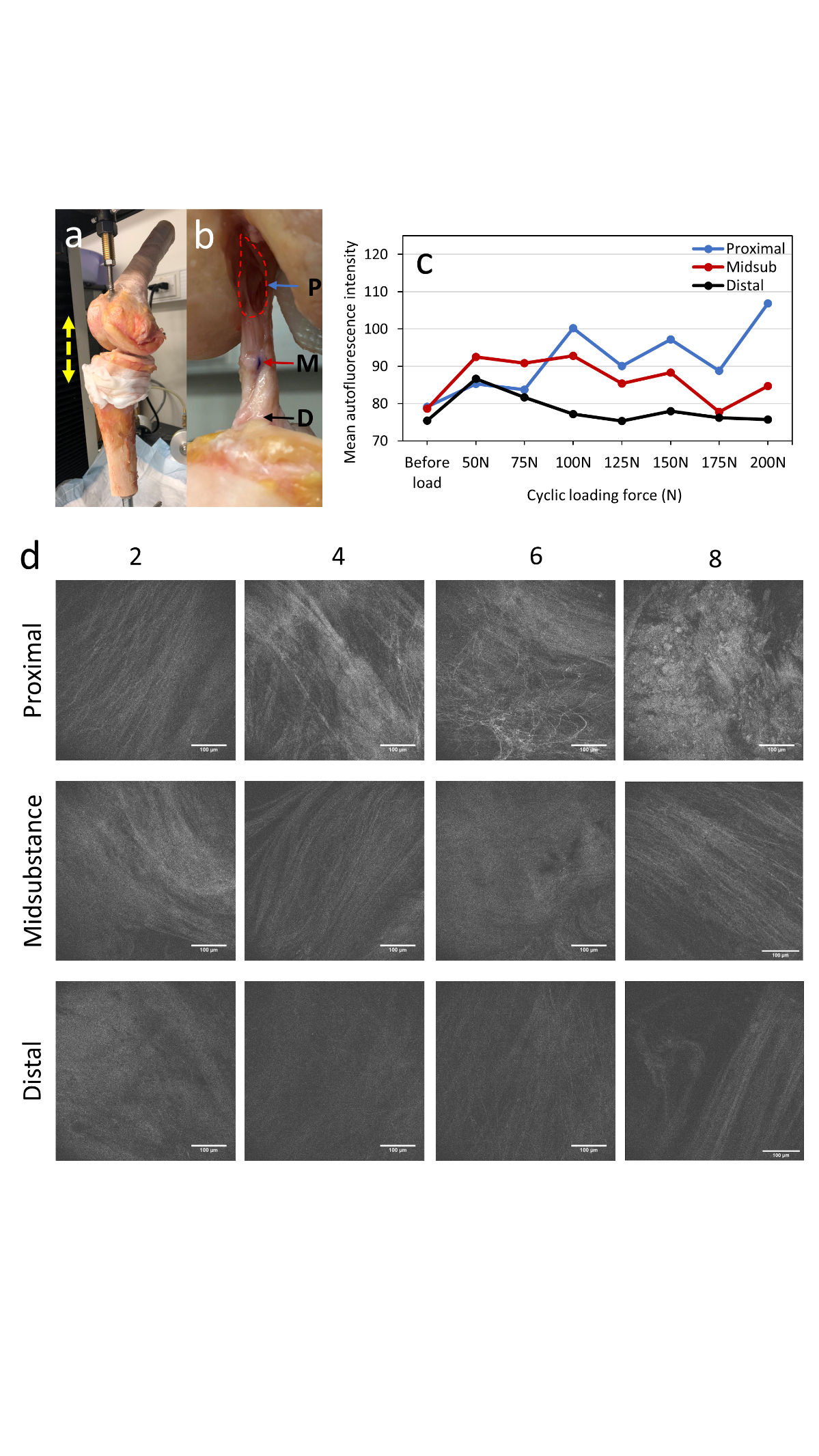
**

**Figure S10**. **Femur – ACL – Tibia complex (FATC) cyclic tensile test of ACL following sequence in Table S2**. a) Cadaver knee with 45° flexion angle in the tensile test device. Femur was exercised up and down (yellow arrow) b) ACL with a partial tear (red dotted circle) at the proximal region during the 11^th^ cycle of the 8^th^ testing sequence with 200 N cyclic load at 20 mm/min. The three regions of AF image acquisition are proximal (P), midsubstance (M) and distal (D). c) The change in mean AF intensity of the image for the three regions exhibit the largest AF signal in the proximal region at the end of the testing sequence, corresponding to the hole. (standard error for each data point range was 20 – 35 not shown). d) CLEM AF images of ACLs during testing sequence number 2, 4, 6 and 8 (point of tear). Scale bar: 100 μm.
